# LncRNAs in tumor metabolic reprogramming and tumor microenvironment remodeling

**DOI:** 10.3389/fimmu.2024.1467151

**Published:** 2024-10-30

**Authors:** Jianhang Jiao, Yangzhi Zhao, Qimei Li, Shunzi Jin, Zhongshan Liu

**Affiliations:** ^1^ Department of Orthopedics, The Second Affiliated Hospital of Jilin University, Changchun, Jilin, China; ^2^ Department of Hematology, The First Hospital of Jilin University, Changchun, China; ^3^ Department of Radiation Oncology, The Second Affiliated Hospital of Jilin University, Changchun, China; ^4^ NHC Key Laboratory of Radiobiology, Jilin University, Changchun, China

**Keywords:** lncRNAs, metabolic reprogramming, tumor microenvironment remodeling, tumor immunity, tumor immunotherapy

## Abstract

The tumor microenvironment (TME) is a complex and dynamic ecosystem composed of tumor cells, immune cells, supporting cells, and the extracellular matrix. Typically, the TME is characterized by an immunosuppressive state. To meet the demands of rapid proliferation, cancer cells undergo metabolic reprogramming, which enhances their biosynthesis and bioenergy supply. Immune cells require similar nutrients for activation and proliferation, leading to competition and immunosuppression within the TME. Additionally, tumor metabolites inhibit immune cell activation and function. Consequently, an immunosuppressed and immune-tolerant TME promotes cancer cell proliferation and metastasis. Long non-coding RNAs (lncRNAs), a category of non-coding RNA longer than 200 nucleotides, regulate tumor metabolic reprogramming by interacting with key enzymes, transporters, and related signaling pathways involved in tumor metabolism. Furthermore, lncRNAs can interact with both cellular and non-cellular components in the TME, thereby facilitating tumor growth, metastasis, drug resistance, and inducing immunosuppression. Recent studies have demonstrated that lncRNAs play a crucial role in reshaping the TME by regulating tumor metabolic reprogramming. In this discussion, we explore the potential mechanisms through which lncRNAs regulate tumor metabolic reprogramming to remodel the TME. Additionally, we examine the prospects of lncRNAs as targets for anti-tumor therapy and as biomarkers for tumor prognosis.

## Graphical Abstract

1. lncRNAs can regulate the metabolic reprogramming process of tumor cells, resulting in increased aerobic glycolysis, lipid metabolism and glutamine metabolism of tumor cells. 2. Tumor metabolic reprogramming can further remodel the tumor microenvironment, resulting in increased lactic acid, decreased PH value, increased cholesterol, free fatty acids and diacylglycerol, as well as decreased glutamine in tumor microenvironment. 3. Tumor microenvironment remodeling induced by tumor metabolic reprogramming can affect immune cell function and form an immunosuppressive microenvironment.

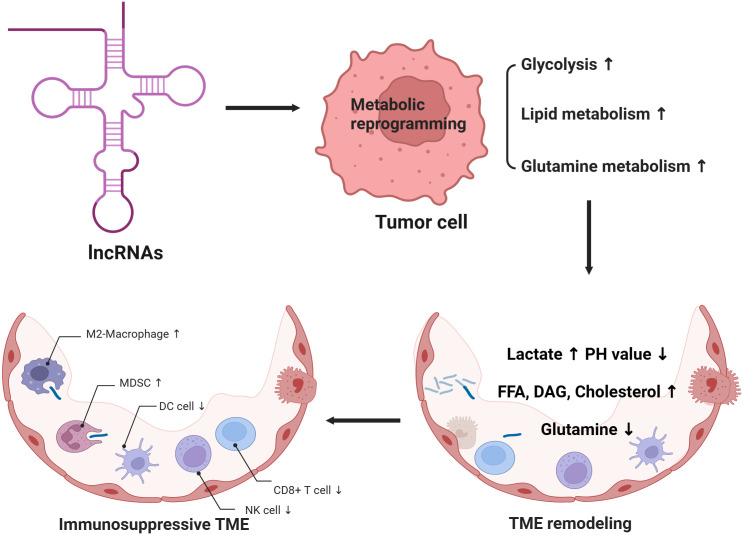

## 1 Introduction

The mortality rate of malignant tumors ranks second among human diseases, which seriously threaten human health (1). Immunotherapy can activate immune cells and enhance antitumor immune responses, changing the treatment mode of tumors. However, most patients either fail to respond to immune checkpoint inhibitors (ICIs) or develop drug resistance. The tumor immune microenvironment plays a pivotal role in tumor immunity and largely determines the effectiveness of immunotherapy. To maintain malignant growth and rapid proliferation of tumors, cancer cells consume a great amount of nutrients, for instance, oxygen, glucose, glutamine, and amino acids, resulting in nutrient deficiency and hypoxia in the tumor microenvironment (TME). As tumor cells compete with immune cells for nutrients in the TME, the activation and proliferation of immune cells are inhibited (2–4). Furthermore, tumor cells can influence immune and stromal cells within the TME, resulting in an immunosuppressive environment that facilitates tumor immune escape. Therefore, understanding how tumor metabolic reprogramming influences the TME can enhance immunotherapy efficacy and identify new therapeutic targets.

Non-coding RNAs (ncRNAs) occupy for approximately 60% of transcriptional output in human cells (5). Although they do not encode proteins, they play indispensable roles at the transcriptional and post-transcriptional levels by combined with DNA, RNA, and proteins to regulate gene expression and protein function. A diverse array of ncRNAs establishes an intricate network that influences numerous biological functions and signaling pathways (5). The disorder of non-coding RNA is closely related to malignant tumors, nervous system diseases, cardiovascular diseases, and autoimmune diseases. Notably, dysregulation of ncRNAs has been associated with all malignancies studied to date and affects all major tumor biomarkers (6, 7). As the most abundant category of non-coding RNAs, long non-coding RNAs (lncRNAs) can influence tumor metabolic reprogramming by regulating key enzymes, transporters, and related signaling pathways in tumor metabolism. Metabolites in the process of tumor metabolic reprogramming, such as lactic acid and free cholesterol, can impair the activation and function of antitumor immune cells, for example, T and NK cells, and promote the polarization of macrophages to the M2 subtype, eventually forming a tumor immunosuppressive microenvironment. Therefore, lncRNAs affect TME remodeling through tumor metabolic reprogramming (8, 9). In this paper, we introduced the role and relationship of lncRNAs in tumor metabolic reprogramming and tumor microenvironment remodeling and summarized the prospects of lncRNAs as future anti-tumor therapeutic targets and prognostic biomarkers.

## 2 Overview of lncRNAs

LncRNAs are an important category of non-coding RNA that are greater than 200 nucleotides in length (10). Based on their localization in the genome, lncRNAs can be classified into sense lncRNAs, antisense lncRNAs, intronic lncRNAs, and intergenic lncRNAs. Sense lncRNAs overlap with the positive strand RNA of neighboring genes, and the transcription direction is the same as that of neighboring genes. Antisense lncRNAs are opposite to sense lncRNAs. Antisense lncRNAs overlap with the negative strand RNA of neighboring genes, and its transcription direction is opposite to that of neighboring genes. Intronic lncRNAs are produced in the intron region within genes by different splicing methods. Intergenic lncRNAs, also known as “lincRNAs,” are located between two genes and usually do not overlap with known protein-coding genes (11, 12). In addition, lncRNAs has strong tissue specificity and cell specificity. In one study, the expression of hundreds of novel lncRNAs was shown to be cell type-dependent (13). Previous investigations have suggested that lncRNAs affect the occurrence and progression of tumors via regulation of the epigenome, gene transcription, or protein translation (14, 15).

**Figure 1:**
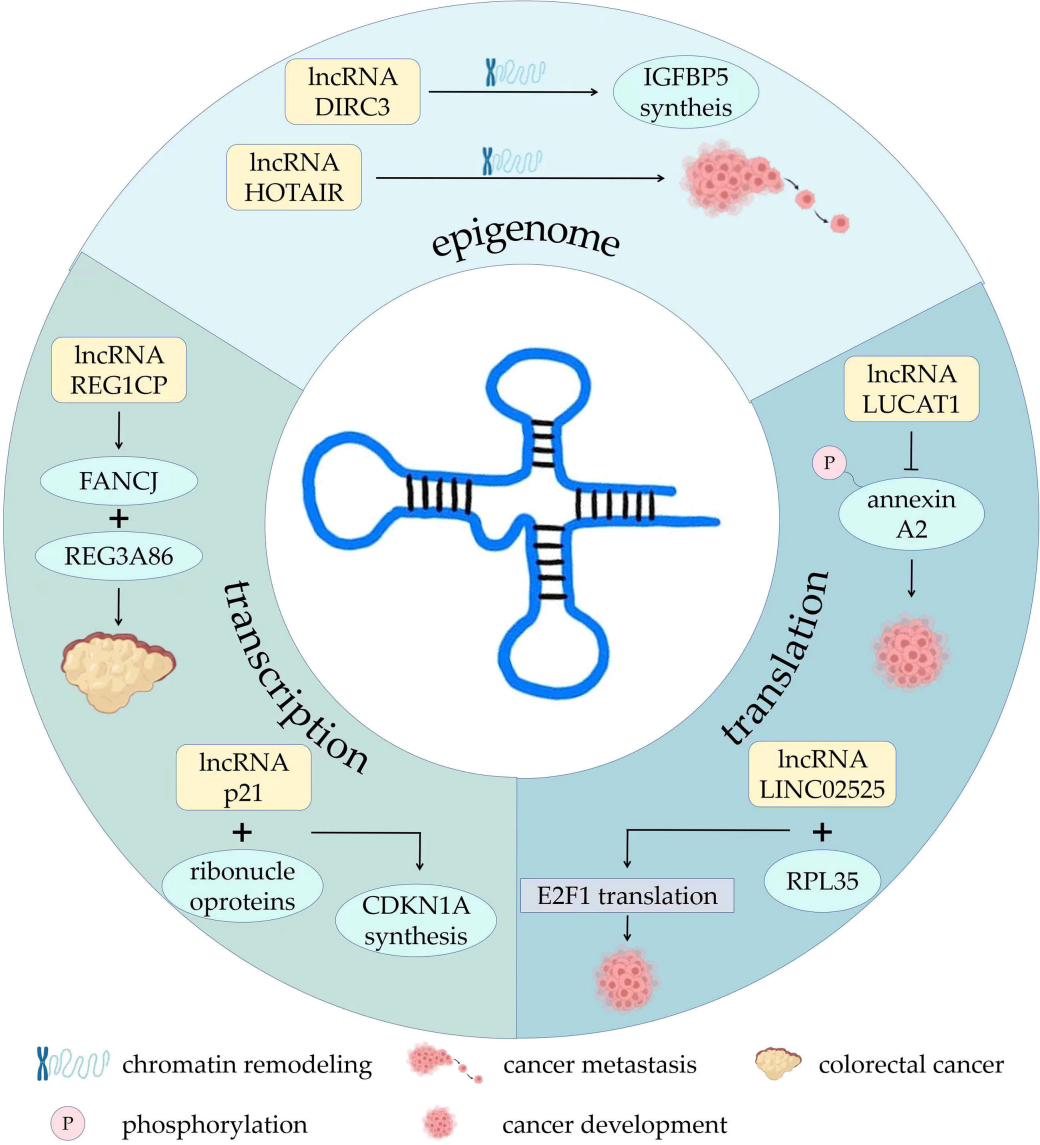
The roles and mechanisms of lncRNAs in development of tumor. AIIT, P, phosphorylation.

Some lncRNAs mediate the recruitment of chromatin remodeling complexes or serve as scaffolds for chromatin remodeling complexes (16, 17). LncRNA HOTAIR had been shown to regulate chromatin remodeling, thereby promoting breast cancer metastasis (18). LncRNA DIRC3 in the nucleus affect the local chromatin structure and activate the transcription of tumor suppressor IGFBP5 (19). LncRNA LINC00261 is involved in phosphorylation of ataxia telangiectasia mutated protein (ATM) and DNA damage of lung adenocarcinoma cells, slowing cancer progression (20). LncRNAs can also activate or repress gene expression by binding to or removing transcription factors (21). Some antisense lncRNAs specifically bind to complementary mRNAs to regulate gene splicing, translation, or degradation at the post-transcriptional level (22). LncRNA REG1CP binds the helicase FANCJ to the promoter of the neighboring gene REG3A86, promoting growth of colorectal cancer (23). LncRNA-p21 can bind to heterogeneous ribonucleoproteins, increasing transcription of neighboring gene CDKN1A and synthesis of P21 protein (24). Other lncRNAs can alter protein localization, regulate protein activity, or serve as components of protein complexes. For example, a HIF-1α anti-sense lncRNA, HIFAL introduces the PKM2/PHD3 complex into the nucleus by binding to heterogeneous nuclear ribonucleoprotein F (hnRNPF) to enhance the production of hypoxia-inducible factor-1 (HIF-1) and promote breast cancer. Therefore, HIFAL is also a target for treatment of breast cancer (25, 26). LncRNA LUCAT1 inhibits phosphorylation of annexin A2, further inhibiting the degradation of ANXA2-S100A10 heterotetramer (AIIT) and promoting cancer development (27). In neuroblastoma, lncRNA LINC02525 interacts with ribosomal protein RPL35. They then specifically activated the translation of E2F1, advancing neuroblastoma (28). There are also lncRNAs that can cleave and produce small RNAs precursors or serve as competitive endogenous RNAs that act as “sponges” for miRNAs (29). Several studies have indicated that lncRNAs play indispensable roles in tumor growth, metastasis, angiogenesis, drug resistance, cell metabolic reprogramming, and the induction of immunosuppression through a variety of complex mechanisms (30–32) (**Figure 1**).

## 3 Metabolic reprogramming of tumors

Body metabolism includes glucose, lipids, amino acids, and the metabolism of other nutrients. The metabolism of various substances affects each other, and metabolic signaling pathways interact with each other to form a complex metabolic network. The metabolic patterns of cancer cells are quite different from those of normal cells. The environment of the cancers has special characteristics, such as hypoxia, acidity, oxidative stress, and nutrient deficiency. To better adapt to this environment, tumor cells must adjust their metabolic pathways to meet the needs of their various life activities, which is called metabolic reprogramming (33). On the one hand, metabolic reprogramming of cancer promotes tumorigenesis by facilitating rapid proliferation, survival, invasion, and metastasis. On the other hand, as the tumor progresses, tumor cells acquire more mutations and alterations, resulting in enhanced metabolic reprogramming, which in turn accelerates tumor growth, proliferation, and development.

**Figure 2:**
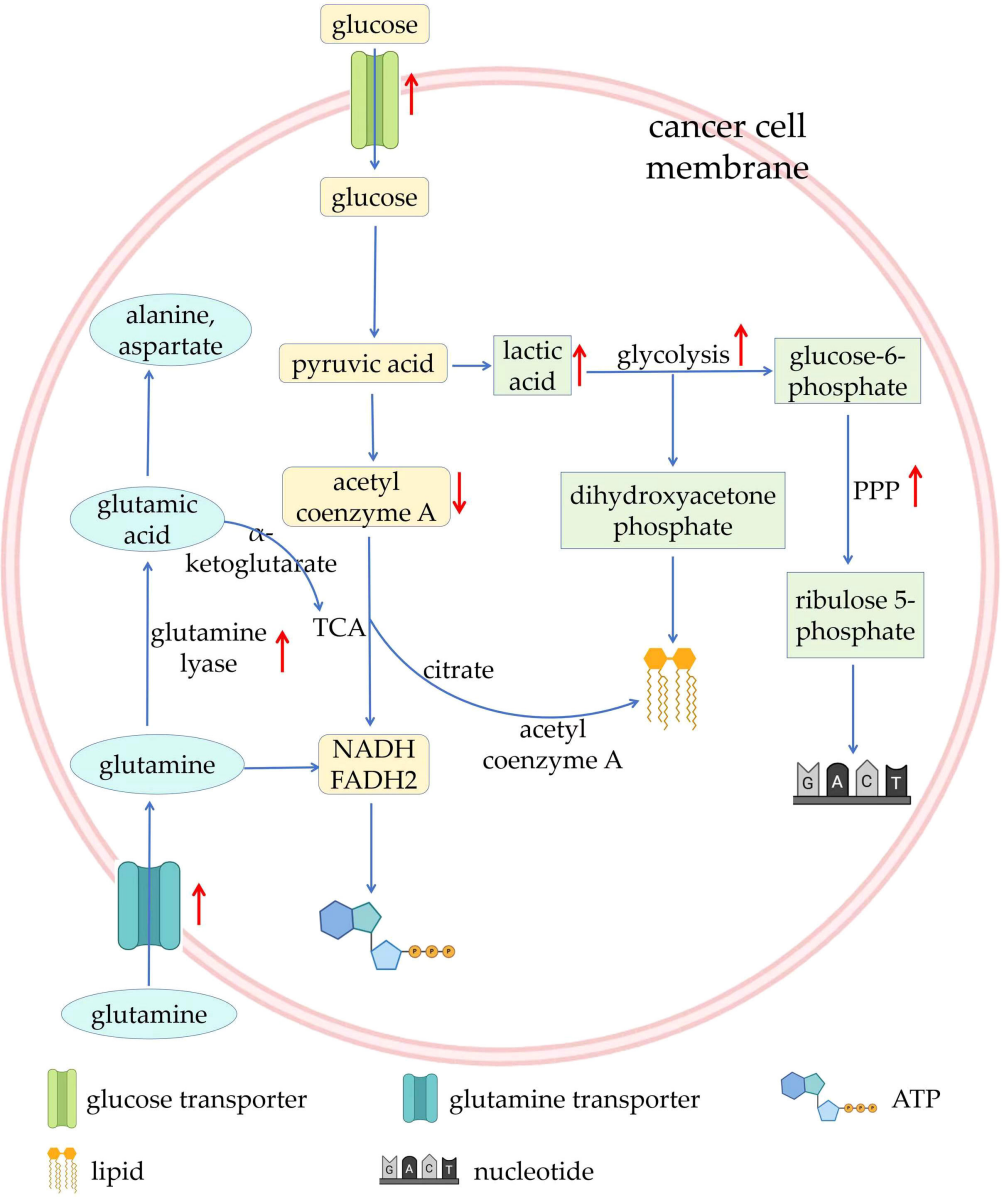
Overview of metabolic reprogramming in tumors. ATP, adenosine triphosphate; PPP, pentose phosphate pathway; TCA, tricarboxylic acid cycle.

Metabolic and functional changes of tumor cells mainly include: 1) Healthy cells rely on mitochondria to oxidize glucose and release energy, while most tumor cells supply themselves with energy through glycolysis, which has a relatively low energy yield. This metabolic property of tumor cells is also known as the Warburg Effect. Since glycolysis has relatively little capacity, cancer cells must maintain their vital activities by upping their glucose intake. Many cancer cells do this by activating glucose transporters. In addition, tumor cells undergoing metabolic reprogramming use transcription factors such as HIF-1α and c-Myc to up-regulate glycolytic enzyme activity, thereby increasing glycolytic efficiency. At the same time, the conversion of glucose to lactate, rather than to pyruvate increases, which means that less pyruvate enters the mitochondria, leading to inhibition of the tricarboxylic acid cycle (TCA) pathway (34, 35); 2) Since pyruvate is overconsumed in the glycolytic pathway, glutamine is used to supplement important metabolites in TCA. As complementary substrate for TCA, glutamine continuously produces NADH, FADH2, and electrons for adenosine triphosphate (ATP) production through oxidative phosphorylation of the mitochondria. Cancer cells depend on c-Myc transcription factor to increase the expression of glutamine transporter and glutamine lyase, leading to increased cellular uptake and utilization of glutamine (36). Glutamine can produce glutamic acid under the catalysis of glutaminase, and glutamic acid can be converted into alanine, aspartate and other amino acids. At the same time, glutamic acid can be used as a substrate to synthesize α-ketoglutaric acid, continuing to provide fuel for TCA (37); 3) In terms of lipid metabolism, in metabolically reprogrammed cancer cells, most acetyl-CoA used for lipid synthesis comes from citrate produced by the TCA in the mitochondria. In the cytoplasm, citrate can be converted back to acetyl-CoA and lipid synthesis occurs (38); 4) anabolism, including nucleotide synthesis, nonessential amino acid synthesis, lipid synthesis, and hexosamine synthesis, is upregulated, which consumes large amount of energy; 5) The pentose phosphate pathway (PPP) is upregulated, which maintains cellular redox homeostasis and downregulates the generation of reactive oxygen species. As a rate-limiting enzyme in the PPP, glucose-6-phosphate dehydrogenase (G6PD) is frequently elevated in human malignancies, which in turn leads to the production of precursors for nucleotide and lipid synthesis (39–41) (**Figure 2**).

## 4 TME

The TME is a complex microenvironment for tumor cells to survive and is made up of tumor cells, stromal cells, fibroblasts, infiltrating immune cells, and the secreted products and extracellular matrix of the corresponding cells. Immune cells are key players in shaping inhibitory microenvironments. In the TME, immune cells have distinct functions compared to normal tissues. For purpose of satisfying the need of rapid proliferation of tumor cells, they undergo metabolic reprogramming, which causes the TME to show ion homeostasis imbalance, acidity, hypoxia, increased lactate, decreased glucose concentration, nutritional competition, and changes in the secretome. Remodeling of the TME induces the metabolic reprogramming of immune cells, which changes their functions. They exhibit an attenuated inflammatory response or enhanced inhibitory function, assisting immune escape from the tumor. Therefore, TME remodeling caused by tumor metabolic reprogramming is the basis for the functional transformation of immune cells and is particularly critical for the proliferation and metastasis of cancer cells.

The most obvious feature of the TME is the constantly changing in its constituents, particularly in the later stages of cancer development. Tumor-derived cytokines, chemokines, and even metabolic conditions (pH, oxygen levels, and nutrients) affect the function of immune cells in TME (42). In the early stages of oncogenesis, the TME is an immune-activated microenvironment with high proinflammatory signals, and immune cells are prone to show a pro-inflammatory phenotype. In the process of cancer growth and metastasis, the TME changes into an immunosuppressive environment step by step, with low oxygen, low pH, low glucose accumulation, high fatty acid accumulation, low amino acid accumulation, high adenosine accumulation, and high lactic acid accumulation, and immune cells are inclined to show an inhibitory phenotype (43). Unlike other microenvironments in the body, TME changes are mainly dominated by tumor cells rather than by the body itself, which is largely out of the control of the body. In this sophisticated, constantly changing, and uncontrollable microenvironment, the roles of various non-tumor cells are also complex and dynamic. For example, immune cells differentiate into subsets with different phenotypes, metabolic characteristics, and functions, which play anti-tumor or pro-tumor roles, and further change the TME through their own metabolism.

### 4.1 Immune in the TME

#### 4.1.1 MDSCs in the TME

Myeloid-derived suppressor cells (MDSCs) in TME often exhibit immunosuppressive properties and can promote tumor progression by inhibiting the function of anti-tumor immune cells. The important mechanism of MDSCs-mediated immunosuppression lies in its metabolic activity. Hypoxia and lactate accumulation in TME lead to increased expression of HIF-1α, which activates the expression of CD39/CD73 on MDSCs, promoting differentiation and proliferation of MDSCs (44, 45). Furthermore, HIF-1α can alleviate MDSCs damage by reducing oxidative stress and ROS production through HIF1α-frataxin signaling (46). At the same time, the acidic environment of TME can enhance the inhibitory function of MDSC on T cells through HIF-1α signaling (44).

#### 4.1.2 DCs in the TME

Dendritic cells (DCs) play an important role in the process of presenting tumor antigens and realizing immune regulation. In TME, bioactive substances secreted by tumor cells can achieve immune escape by inhibiting recruitment and maturation of DCs (47). For example, granulocyte colony-stimulating factors (G-CSF) secreted by breast cancer cells lead to directional differentiation of bone marrow precursor cells to MDSCs instead of DCs, and inhibit maturation of DCs by down-regulating CD80 and CD86 (48). GRP78 secreted by breast cancer cells induces differentiation of DCs into regulatory DCs (DCreg), and TGF-β secreted by DCreg significantly inhibits the killing activity of NK cells (49). Cytotoxic T (CD8^+^T) cells and natural killer (NK) cells secrete toxic substances to kill tumor cells or directly engulf tumor cells, forming a strong defense line in anti-tumor immunity. However, in the immunosuppressive microenvironment, the functions of both CD8^+^T cells and NK cells are inhibited. For example, exosomes secreted by melanoma induce apoptosis of CD8^+^T cells in lymph nodes and proliferation of CD8+T cells is significantly inhibited by CXCL1 and CXCL2 secreted by ovarian cancer cells (50, 51). Ovarian cancer cells overexpress the immune checkpoint B7-H3, which promotes glycolysis and results in dysfunction of NK cells (52).

#### 4.1.3 ILCs in the TME

Innate lymphoid cells (ILCs) are also an important component of the tumor microenvironment. ILCs are a group of innate immune cells that do not express antigen-specific receptors. Based on their cytokine production profiles and transcription factor expression, they can be classified into different subgroups (ILC1, ILC2, ILC3). Research has shown that ILCs have both pro-tumor and anti-tumor effects within the tumor microenvironment (53). When performing immune surveillance functions, ILCs can recognize tumor cells and regulate the tumor microenvironment by secreting cytokines, which inhibits tumor growth. However, interactions with other immune cells, such as T cells and macrophages, allow ILCs to promote angiogenesis, tissue remodeling, and immune suppression, thereby supporting tumor growth.

#### 4.1.4 Pericytes in the TME

Pericytes are also an important component of the immune microenvironment, participating in angiogenesis and stabilization. They can regulate vascular permeability, secrete cytokines and chemokines that affect the infiltration of tumor cells and immune cells in the tumor microenvironment, and thus help modulate the immune response within this environment. Their influence may promote or inhibit tumor growth by affecting the migration and function of immune cells (54). Therefore, the metabolic activities of tumor cells and immune cells form a sophisticated and complex interaction network with TME.

#### 4.1.5 Dual-function immune cells in the TME

In addition, there are some dual-function immune cells in TME, such as neutrophils and macrophages. N1 neutrophils and M1 macrophages work against tumors, while N2 neutrophils and M2 macrophages promote tumor (55). Some studies had found that ETS1 exosomes secreted by ovarian cancer cells induce the polarization of M2 macrophages, which overexpress CD163, IL-10, CCL2, CXCL5 and other immunosuppressive factors, and significantly stimulate the migration of ovarian cancer cells (56). The pluripotent factor Lin28B secreted by breast cancer cells induce neutrophils to transform into N2 phenotype, significantly inhibiting proliferation of CD4^+^ and CD8^+^T cells (57) (**Table 1**).

**Table 1:** The state and mechanism of various immune cells in TME.

Immune Cells	State	Tumor Type	Mechanism
MDSCs	activation	ovarian cancer	HIF-1α activates the expression of CD39/CD73 on MDSCs.
DCs	inhibition	breast cancer	G-CSF down-regulates CD80 and CD86 of DCs.
	differentiation into DCreg	breast cancer	GRP78 induces the differentiation into DCreg.
CD8^+^T cells	inhibition	melanoma	Exosomes induce apoptosis of CD8^+^T cells.
		ovarian cancer	CXCL1 and CXCL2 inhibit proliferation of CD8^+^T cells.
NK cells	inhibition	ovarian cancer	B7-H3, an immune checkpoint promotes dysfunction of NK cells.
Neutrophils	polarization of N2 neutrophils	ovarian cancer	ETS1 exosomes induce the polarization of N2 neutrophils.
Macrophages	polarization of M2 macrophages	breast cancer	Lin28B induce the polarization of M2 macrophages.

CD8^+^T cells, cytotoxic T cells; DCreg, regulatory dendritic cells; DCs, dendritic cells; G-CSF, granulocyte colony stimulating factors; HIF-1α, hypoxic inducible factor 1-α; MDSCs, myeloid-derived suppressor cells; NK cells, natural killer cells.

## 5 LncRNAs bridges metabolic reprogramming and TME remodeling

**Figure 3:**
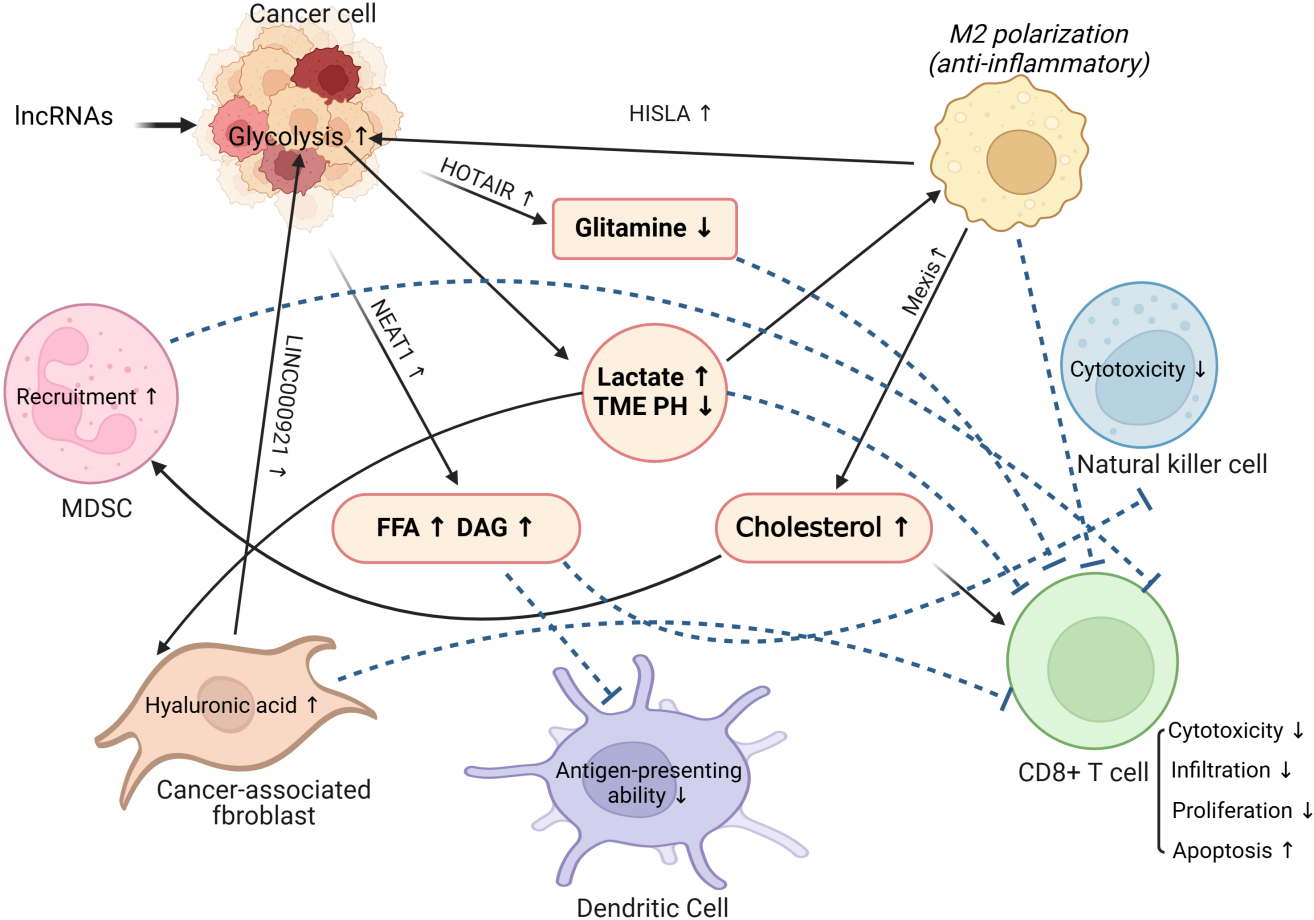
The role of lncRNAs in linking tumor metabolic reprogramming and tumor microenvironment remodeling. MDSC, Myeloid-derived suppressor cells; TME, Tumor microenvironment; FFA, Free fatty acids; DAG, Diacylglycerol.

It is not only tumor cells that require macronutrients and energy to meet their own needs for proliferation and metastasis. For purpose of accomplishing the proliferation, differentiation, and effector functions of various immune cells, the immune system requires a large number of similar nutrients for biosynthesis. In addition, the metabolic modes of immune cells in the activated and effector states are markedly distinguishable from those in the resting state. For example, the metabolism of naïve T cells is usually quiescent, mainly through oxidative phosphorylation to produce energy. Once differentiated into effector T-cells, they lean primarily upon glycolysis for energy production (58). Neutrophils rely mainly on aerobic glycolysis and the pentose phosphate pathway to provide energy. Glycolysis is a primary metabolic pathway in activated B lymphocytes (59). However, the oxidative phosphorylation of fatty acids is a major metabolic pattern in both regulatory T cells and M2 macrophages (60). After the tumor initiates metabolic reprogramming, the function of the immune system is impaired by the competitive uptake of nutrients, for instance, glucose, fatty acids, glutamine, and amino acids. Investigations have shown that cancer cells can compete for glucose uptake and enhance glycolysis to inhibit the function of tumor-infiltrating lymphocytes (TILs), limit glucose consumption by TILs, and ultimately result in T cell exhaustion and tumor immune evasion. Notably, not only changes in tumor metabolic patterns but also metabolites from tumor reprogramming can affect the phenotype and function of immune cells. The accumulation of lactic acid can inhibit the function of T and NK cells. Moreover, lactic acid upregulates the expression of PD-L1 on the surface of tumor cells and mediates T cell exhaustion by binding to the PD-1 receptor on the surface of T cells (61). In addition, reprogramming of tumor lipid metabolism can lead to increased cholesterol levels in the TME, which can upregulate the expression of inhibitory immune checkpoints, such as PD-1 and LAG-3, on the surface of T cells, leading to an increase in the number of exhausted T cells. Similarly, immune cells become less functional and proliferative and are more susceptible to apoptosis (62) (**Figure 3**).

Furthermore, existing research has confirmed that lncRNAs are closely related to the innate immune system (63). The innate immune system serves as the first line of defense against pathogens and relies on a surveillance system involving neutrophils, macrophages, natural killer cells, and dendritic cells. LncRNAs have been shown to function through modular domains, regulating gene expression and modulating pathogen response pathways by interacting with chromatin, RNA, and proteins (64).

They can engage in T cell development, activation, differentiation, function, and cancer immunology by binding specifically to the epidermal growth factor receptor (EGFR) or inducing the expression of cell surface phenotypes, as well as suppressing Treg-mediated immune evasion in hepatoma carcinoma cells (HCCs) (65, 66). Additionally, lncRNAs play a critical role in macrophage differentiation, recruitment, polarization, and functionality. They regulate macrophage differentiation by modulating ACVR1B (a key factor in macrophage differentiation) to activate the TGF-β pathway (67), or by influencing the calcium-dependent kinase PNCK triggered by Ca2+, which leads to macrophage recruitment and angiogenesis. Similarly, lncRNAs are involved in the reprogramming of normal fibroblasts (NF) to cancer-associated fibroblasts (CAFs). They participate in this process through exosomes delivery, gene knockout, and inhibition of autophagy-lysosomal degradation, which further modulates the metabolism and chemotherapy resistance of CAFs (68, 69).

Latest researches have indicated that lncRNAs upregulate the expression of key enzymes and transporters in tumor metabolic reprogramming through a variety of mechanisms and regulate related signaling pathways, eventually leading to changes in the TME (70–75) (**Table 2**). Therefore, lncRNAs may reprogram the tumor immune microenvironment by regulating tumor metabolism.

**Table 2:** The lncRNAs in the network of cancer metabolism.

Effects	LncRNAs	Targets	Tumor Type
Glycolysis ↑ Lactate ↑	HOTAIR (76)	GLUT1 ↑	Hepatoma carcinoma
	ANRIL (70)		Nasopharyngeal carcinoma
	lnc-p23154 (75)		Bladder cancer
	NBR2 (77)		**-**
	MACC1-AS1 (78)		Gastric cancer
	LINC00174 (79)		Glioma
	LINC00346 (80)		Breast cancer
	CamK-A (81)	GLUT3 ↑	Multiple cancers
	CRNDE (82)	GLUT4 ↑	Colorectal cancer
	UCA1 (83)	HK2 ↑	Bladder cancer
	PVT1 (84)		Osteosarcoma
	TUG1 (85)		Hepatoma carcinoma
	LncRNA H19 (86)	PKM2 ↑	Hepatoma carcinoma
	LncRNA 020978 (87)		Non-small cell lung cancer
	LINC00092 (71)	PFKFB2 ↑	Ovarian cancer
	GLCC1 (88)	c-Myc ↑	Colorectal cancer
	FILNC1 (89)		Renal cancer
	PDIA3P (90)		Multiple myeloma
	LincRNA-P21 (91)	HIF-1α Pathway ↑	Ovarian cancer
Glycolysis ↓ Lactate ↓	GAS5 (92)	G-6-PD, PckA ↓	Multiple cancers
	NEF (93)	GLUT1 ↓	Non-small cell lung cancer
Lipid metabolism ↑ DAG, FFA ↑	HULC (94)	PPARA, ACSL1 ↑	Hepatoma carcinoma
	NEAT1 (95)	ATGL ↑	Hepatoma carcinoma
Cholesterol efflux ↑	LncRNA MeXis (96)	ABCA1 ↑	**-**
	LincRNA-DYNLRB2-2 (97)	ABCA1 ↑	**-**
Glutamine metabolism ↑, Glutamine ↓	HOTAIR (98)	GLS ↑	Glioma

↑, Increased; ↓, Decreased.

### 5.1 LncRNA in glucose metabolism and TME

Typically, normal human cells consume glucose and generate an ATP to provide energy to the body through oxidative phosphorylation under aerobic conditions. Interestingly, under aerobic conditions, tumor cells meet their energy requirements through glycolysis, an effect known as the “Warburg Effect” (99). Although aerobic glycolysis produces less ATP than does oxidative phosphorylation, it also produces less reactive oxygen species, which can induce apoptosis in cancer cells (100, 101). LncRNA influences tumor cells by affecting metabolic enzymes and signaling pathways, thus reprogramming their metabolic processes to preferentially convert glucose into lactate, regulating cancer growth, maintenance, and progression (102, 103). In addition, lactate, acetyl-CoA, and ribose generated during glucose metabolism can provide a favorable environment for rapid tumor proliferation and metastasis (104, 105). Five lncRNAs, including MIR4435-2HG, AC078846.1, AL157392.3, AP001273.1 and RAD51-AS1 significantly upregulated in tumors could activate tumor glycolytic metabolic pathways, and were strongly associated with high levels of poor prognosis-related molecules. In particular, the expression of lncRNA MIR4435-2HG was closely related to the high infiltration of M2 macrophages in hepatocellular carcinoma. The finding of these lncRNAs provides new ideas for the establishment of prognostic assessment models for tumor patients and the development of new therapeutic approaches (106–109). Therefore, alterations in glucose metabolism in tumors play a crucial role in their occurrence and development.

Several studies have verified that many lncRNAs affect glucose transporters, various key enzymes, and signaling pathways involved in glucose metabolism to regulate the reprogramming of glucose metabolism in tumors. For instance, the most studied lncRNA involved in genome modification (110), HOTAIR, which is transcribed in the reverse direction of HOXC and represses HOXD transcription by recruiting PRCI in fibroblasts (111), is commonly expressed in HCCs and accelerates cell proliferation by regulating glucose metabolism. Generally, HOTAIR upregulates GLUT1 expression via activating the mTOR signaling pathway and directly binds to HCCs to promote cell proliferation (76). Similarly, ANRIL, as well as UCA1, by activating mTOR signaling pathway and upregulating GLUT1 are involved in glucose metabolism reprogramming in nasopharyngeal carcinoma cells and bladder cancer cells, respectively (70, 83). Additionally, several studies have shown that lnc-p23154, NBR2, MACC1-AS1, LINC00174, and LINC00346 upregulate GLUT1 expression via different mechanisms, which enhance tumor glycolysis (75, 77–80). In contrast, the expression of lncRNA NEF in non-small cell lung cancer (NSCLC) is often decreased, resulting in the downregulation of GLUT1 expression and decreased glycolysis in tumor cells. Thus, NEF negatively regulates tumor progression in NSCLC (93). In addition, CamK-A and CRNDE increase glucose uptake in tumor cells by upregulating the expression of GLUT3 and GLUT4, respectively, to promote aerobic glycolysis (81, 82).

Apart from regulating glucose uptake, lncRNAs can interact with key enzymes and related glycolytic pathways to regulate glucose metabolism reprogramming in tumors. Hexokinase, phosphofructokinase, and pyruvate kinase are the key enzymes in glycolysis (112). UCA1, PVT1, and TUG1 upregulate hexokinase 2 (HK2) expression by interacting with miR-203, miR-497/miR-143, and miR-455-3p, respectively, to boost glycolysis (73, 84, 85, 113–116). LncRNA H19 can upregulate pyruvate kinase M2 (PKM2) expression to promote glycolysis. In addition, lncRNA H19 promotes lactate production through the miR-519D-3p/lactate dehydrogenase A signal axis (86, 117). The lncRNA AC020978 can also directly interact with PKM2 to facilitate glycolysis, proliferation, and invasion of cancer cells (87). Additionally, LINC00092 directly interacts with fructose-2,6-bisphosphatase (PFKFB2) to promote ovarian cancer metastasis by enhancing glycolysis and maintaining CAFs local support functions (71). In contrast, as a negative regulator, GAS5 suppresses glycolysis and tumor cell proliferation by inhibiting glucose 6-phosphate dehydrogenease and phosphoenolpyruvate carboxykinase expression (92). GLCC1, FILNC1, PDIA3P, and other lncRNAs regulate glycolysis by interacting with or regulating the expression of the transcription factor c-Myc (88–90). Besides, LincRNA-P21 and lncRNA AC020978 promote glycolysis by involving the HIF-1α pathway (87, 91).

A great deal of lactate is a significant product of aerobic glycolysis in tumors. Lactate plays an indispensable role in the reprogramming of tumor glucose metabolism and the remodeling of the TME. Elevated lactate concentrations both directly hinder the function of TILs and NK cells, while additionally diminishing their activity, consequently remodeling an immunosuppressive TME (61). Besides, lactate can activate GPR81 receptor on macrophages to lessen inflammasome activation and the generation of pro-inflammatory cytokines, such as IL-6 and TNF-α (118, 119). Moreover, the accumulation of lactate and the decrease in pH value in TME can induce the phenotype of immune cells in TME to be polarized toward immunosuppression and immune tolerance (120). For instance, lactate induces macrophages to polarize to the M2 subtype, sequentially producing the immunosuppressive factor IL-10 (121). Importantly, lncRNAs secreted by extracellular vesicles from tumor-associated macrophages (TAMs), HISLA, can reduce the hydroxylation and degradation of HIF-1α via blocking the interaction between PHD2 and HIF-1α, which promotes glycolysis and lactate accumulation in tumor cells. Conversely, the glycolytic product, lactate upregulates the expression of HISLA in TAMs, constituting a feedforward loop between cancer cells and TAMs (122).

CAFs not only form the matrix components of the TME but are also important regulatory factors in the TME. Large amounts of lactate in the TME would promote the generation of hyaluronic acid in CAFs, which facilitates tumor invasion and metastasis (123). Furthermore, in the presence of high levels of lactate in the TME, CAFs decrease the percentage of Th1 cell subsets via SIRT1-mediated deacetylation/degradation of the T-bet transcription factor. Besides, CAFs can promote the polarization of naive T cells to Treg cells by upregulating the expression of NF-kB and FoxP3 (124). Intriguingly, studies have shown that CAFs upregulate LINC00092 to promote aerobic glycolysis and lactate generation in ovarian cancer, which further reshapes the TME (71). Therefore, tumor metabolic reprogramming and TME remodeling are interlinked and involve complex regulatory networks. LncRNAs can act as upstream regulators and downstream effectors to promote tumor glycolysis and lactate secretion and mediate the remodeling of the immune microenvironment. Finally, the immune system progresses towards immunosuppression and immune tolerance.

### 5.2 LncRNA in lipid metabolism and TME

Apart from abnormal glucose metabolism, abnormal lipid metabolism is involved in tumor metabolic reprogramming (125). Characteristic changes in lipid metabolism in tumors include *de novo* lipid synthesis, lipid storage, and the esterification of cholesterol to free cholesterol. Abnormal lipid metabolism in tumor cells provides an important energy source and material for tumor cell proliferation and metastasis. Lipid metabolism plays a vital role in maintaining immune cell development and function in TME (126). Treg cells rely on lipid oxidation for energy rather than glycolysis because they have significantly few glucose transporters on their surfaces. Therefore, the addition of exogenous fatty acids can promote the production of Treg and inhibit the formation of effector T cells (126). T cells synthesize lipids as an energy source through the fatty acid oxidation catabolic pathway and induce lipid biosynthesis through *de novo* fatty acids (127). Additionally, fatty acids are indispensable for the memory and cytotoxic functions of CD8^+^ T cells (128). As vital regulators of fatty acid and fat synthesis, phospholipid metabolism, and transport, a large number of lncRNAs form a complex regulatory network through a variety of mechanisms and pathways to induce lipid reprogramming and immune microenvironment remodeling in tumor cells (129).

Studies have shown that HULC can induce the methylation of CpG islands in the promoter region of miR-9, resulting in an increase in peroxisome proliferation activated receptor alpha (PPARα) and acyl CoA synthetase 1 (ACSL1) level, which in turn leads to the accumulation of triglycerides and cholesterol in HCCs. The product of ACSL1 can activate the transcription factor RXRA to augment HULC expression, which leads to an increase in lipids in the TME (94). Similarly, NEAT1 can regulate adipose triglyceride lipase (ATGL) expression by binding to miR-124-3p in HCCs, leading to enhanced lipolysis and increased fatty acids and diglyceride levels (95). Recent research has shown that lncRNAs are enriched in both mitochondria and the cell nucleus, playing a direct or indirect role in significant metabolic processes such as fatty acid metabolism. For instance, circNFIX participates in fatty acid metabolism in breast cancer by regulating the expression of MMP9, while the cis gene ACACB modulates the rate-limiting enzyme in fatty acid oxidation, thereby controlling fatty acid metabolism (130, 131).Notably, lipid accumulation in the TME can further impair the function of a variety of immune cells and induce a tumor-immunosuppressive microenvironment. For example, increased lipid levels decrease the antigen presenting capacity of dendritic cells (132). Likewise, in the presence of PPARα-mediated abnormal fatty acids elevation, the function of NK cell-tumor synaptic transport is inhibited, resulting in poor immune surveillance (133). In addition to reshaping the TME by regulating tumor cell lipid metabolism, NATE1 directly inhibits immune cell function. For instance, NEAT1 inhibits the antitumor immune function of cytotoxic T cells by downregulating the cyclic GMP-AMP stimulatory expression of the interferon gene (72). NEAT1 also regulates dendritic cell and macrophage functions and phenotypes by inducing NLRP3 inflammasomes (74, 134). Low NEAT1 expression levels are associated with high CD8^+^ T cell infiltration in tumor tissues (72). Moreover, NEAT1 interacts with miR-214 to regulate B7-H3 expression in multiple myeloma, leading to the polarization of macrophages to the M2 subtype, thereby inducing the formation of a tumor-immunosuppressive microenvironment (135).

In the reprogramming of tumor lipid metabolism, cholesterol and its derivatives also play vital roles in inducing a tumor immunosuppressive microenvironment (136). Previous studies have reported that cholesterol derived from the TME increases the expression of CD36 and fatty acid uptake by CD8^+^ T cells. Superabundant fatty acid intake can induce lipid peroxidation and ferroptosis of CD8^+^ T cells, which impairs antitumor immune function (137). In addition, the cholesterol hydroxylation products 25-hydroxycholesterol and 27-hydroxycholesterol can increase MDSC infiltration and reduce CD8+T cell numbers in the TME, inducing tumor immunosuppression (138, 139). Under conditions of LXR activation, lncRNA MeXis can enhance ABCA1 expression to facilitate cholesterol efflux (96). Similarly, the LincRNA-DYNLRB2-2 upregulates low density lipoprotein-induced ABCA1 expression, leading to increased cholesterol efflux (97). Cholesterol efflux increases the levels of cholesterol and its derivatives in the TME, thereby inducing TME immunosuppression and immune tolerance.

Sphingolipids, especially ceramides are also critical for TME. By regulating sphingolipids, lncRNAs can further regulate biological behaviors of tumors, such as the occurrence and development. For example, lncRNA ST3Gal6 antisense 1 (ST3Gal6-AS1) promotes metabolism of ceramide in osteosarcoma cells, then promoting the polarization of M2 macrophages, leading to higher level of IL-6 and IL-10 and causing immune escape in osteosarcoma (140, 141). Among sphingolipids-associated lncRNAs, lncRNA ceramide synthase 6 antisense RNA 1 (CERS6-AS1) is the most studied. In May 2022, Zhao et al. pointed out that lncRNA CERS6-AS1 upregulated ceramide and spectrin beta, non-erythrocytic 2 (SPTBN2), thereby promoting the malignant phenotype of colorectal cancer (142). Subsequently, the role of lncRNA CERS6-AS1 in various cancers was studied. LncRNA CERS6-AS1 promotes transcription of ceramide and the proliferation of pancreatic cancer cells, breast cancer cells and cervical cancer cells (143–145).

Overall, lncRNAs and their signaling pathways constitute a complex network that precisely regulates tumor lipid metabolism. The TME, including immune cells, is also affected. An exhaustive understanding of the mechanisms of the entire regulatory network can help us identify new approaches for antitumor therapy.

### 5.3 LncRNA in amino acid and TME

Amino acids are key nutrients for cancer cells, and the regulation of their metabolism has become a focal point for many studies. These investigations focus on changes in key enzyme genes involved in amino acid metabolism, as well as epigenetic modifications, transcription, translation, and post-translational modifications. Disruption of amino acid metabolism is one of the critical features of cancer metabolism. Amino acid metabolism plays a vital role in cancer cells, including energy production, nucleotide synthesis, and maintaining redox homeostasis. To endure in this nutrient-constrained milieu, cancer cells employ a diverse array of trophic sensing and metabolic mechanisms, dynamically reprogramming their metabolic pathways (146, 147). Accumulating evidence underscores the pivotal role of lncRNAs in these adaptive metabolic responses, particularly in instances where numerous ncRNAs undergo dysregulation under conditions of nutrient scarcity (148). These lncRNAs profoundly influence metabolic alterations and contribute to the malignant transformation of cancer cells (149). Also of interest is the short peptide coding capacity of lncRNAs, despite their limited coding capacity was generally recognized, which has also been confirmed in several recent studies in part (150). Especially the capacity to regulate fatty acid metabolism and redox processes, which can be achieved through peptide-coding lncRNAs, subsequently diminishing the viability and migration of cancer cells (151).

#### 5.3.1 Glutamine metabolism

Glutamine metabolism plays an indispensable role in tumor metabolic reprogramming by providing a carbon source for the tricarboxylic acid cycle and a nitrogen source for amino acid and nucleotide synthesis to meet the demand for rapid tumor cell proliferation. Liu et al. found that lncRNA HOTAIR was abnormally upregulated in glioma, and its content was negatively correlated with miR-126-5p. As a competitive endogenous RNA of miR-126-5p, upregulated HOTAIR led to decreasing expression of glutaminase (GLS), thereby inhibiting glutamine metabolism and enhancing the malignancy of glioma. Understanding the regulatory mechanisms of the HOTAIR/miR-126/GLS axis in gliomas could promote novel treatments for this disease (98). Moreover, HOTAIR also promotes the secretion of CCL2, which recruits TAMs and MDSCs to the TME to induce tumor immunosuppression (152). Upregulation of GLS expression promotes glutamine depletion in tumor cells, leading to HIF-1α activation and IL-23 secretion by TAMs, which can inhibit the tumor-killing effect of cytotoxic lymphocytes (153). Additionally, high glutamine consumption promotes glutamate excretion. Therefore, the metabolism of glutamine can be affected by lncRNA and GLS, thus playing an important role in tumor metabolic reprogramming.

#### 5.3.2 Serine metabolism

Serine is another non-essential amino acid that plays a role in nucleotide synthesis, oxidative stress responses, the TCA cycle, and various other metabolic processes. It can be sourced from extracellular uptake or synthesized through the serine synthesis pathway. The expression of lncRNA MEG8 shows a positive correlation with PSAT1 expression and serine synthesis, with PSAT1 functioning as a competing endogenous RNA (ceRNA) that interacts with miR-15a-5p and miR-15b-5p (154). Interestingly, lncRNAs can influence serine metabolism by modulating the expression of SHMT2 in various types of cancer. In lung cancer, the targets of miR-615-5p include IGF2, SHMT2, and AKT2. LncRNA Gm15290 interacts with miR-615-5p and exhibits a negative correlation with its levels (155). LncRNAs are essential in regulating various enzymes within the serine synthesis pathway and affect tumor progression by modulating serine metabolism. Consequently, further investigation into the regulatory roles of lncRNAs on serine metabolism is of great significance.

#### 5.3.3 Aspartate metabolism

Aspartic acid, one of the amino acids present in the lowest concentrations in the blood, is crucial for protein and nucleotide synthesis, significantly supporting cell growth. The impact of lncRNAs on aspartic acid metabolism primarily involves the regulation of GOT expression. GOT is an essential enzyme linked to both aspartic acid and carbohydrate metabolism, and it also facilitates cancer cell proliferation by helping to maintain redox balance. LncRNA NEAT1 can modulate the expression of GOT1 and the transferrin receptor (TFRC) during ferroptosis (156). Silencing lncRNA-ACOD1 markedly decreases macrophage infections by vesicular stomatitis virus (VSV), vaccinia virus (VACV), and herpes simplex virus type 1 (HSV-1). This indicates a strong association between lncRNAs and aspartic acid metabolism, suggesting that these lncRNAs could be valuable in improving tumor immunotherapy.

Several studies have indicated that Amino acid metabolism is important for tumorigenesis and tumor immunity. However, studies on the regulation of Amino acid metabolism by lncRNAs in tumors are limited. lncRNAs related to Amino acid metabolism are promising metabolic targets for cancer treatment. Future researches should be concentrated on the mechanism by which lncRNAs regulate Amino acid metabolism and immune microenvironment remodeling and identify clinically meaningful targets for anti-tumor molecular targeted therapy.

## 6 Future clinical applications of lncRNA

It has been shown that TAMs secrete lncRNA HISLA via extracellular vesicles. HISLA can reduce the hydroxylation and degradation of HIF-1α via blocking the interaction between PHD2 and HIF-1α, which promotes glycolysis and lactate accumulation in tumor cells. Accumulated lactic acid can further prompt TAMs to secrete HISLA. Thus, HISLA silencing mediated by RNA interference has great potential to interrupt tumor glucose metabolic reprogramming, which will further weaken TME immunosuppression induced by tumor metabolic reprogramming and help restore immune cell function. The use of aptamer-siRNA chimeras to mediate specific HISLA knockdown in TAMs to abort tumor glucose metabolism remodeling and restore antitumor immune functions warrants further investigation. Similarly, lncRNA HIFAL can promote HIF-1α transactivation complex assembly and glycolytic metabolism. Inhibition of HIFAL can weaken tumor glycolysis and reduce the tumor energy supply and glycolytic products, weakening the inhibitory effect of tumor metabolic reprogramming on immune cells (122). This is a potential new target for antitumor therapy.

In addition to lncRNAs involved in regulating tumor metabolism, the immune checkpoint signaling axis regulates tumor metabolic reprogramming. PD-L1 expression in cancer cells can activate the Akt-mTOR signaling axis, enhance glycolysis, prompt glucose competition between the tumor and T cells, and increase lactate production. Notably, lactate accumulation, glucose stress, and PH value decrease in the TME also upregulate PD-1 expression, which promotes tumor immunosuppression and immune evasion (61). Immune checkpoint inhibitors inhibit glycolysis to a certain extent and weaken immune suppression; however, the overall therapeutic efficiency of immunotherapy remains limited. Whether anti-lncRNA-targeted metabolic therapy combined with immunotherapy can further improve the effects of tumor treatment and enhance antitumor immunity is worth exploring in the future. Theoretically, the inhibition of dysregulated lncRNA-mediated reprogramming of tumor metabolism could restore its suppressive effects on immune cells. However, it is undeniable that, given the physiological situation, these metabolic processes also contribute to the activation of the immune system, and the interruption of these metabolic processes will also influence the activation of immune cells and anti-tumor immunity. Therefore, identifying the best method to accurately target disordered tumor metabolic reprogramming is a direction for our future research.

Targeting lncRNAs operate precise biological functions through various mechanisms, notably as guides, scaffolds, and decoy molecules, among others. A profound understanding of these multifaceted roles is crucial for the development of highly efficient targeting strategies (157). However, the clinical implementation of therapeutic targeting of lncRNAs has been constrained by several unresolved issues. Firstly, the low level of conservation among species for lncRNAs poses a significant hindrance to numerous research models. Furthermore, the subcellular localization of lncRNAs varies significantly between human and mouse embryonic stem cells, and their stability also differs markedly between the two species (158). This implies the potential irrelevance of findings from other organisms to human clinical contexts consequently (159). Moreover, lncRNAs enable targeting multiple genes, which increases the potential probability of off-target effects when attempting to target them. It is imperative that clinical trials meticulously consider both the likelihood and the extent of these effects, aiming to mitigate them in the safest manner possible. Notably, these intricacies may not be fully elucidated through animal studies alone is a formidable challenge in lncRNA-targeting strategies (160). To mitigate the aforementioned issues, incorporating transcriptome-wide association study (TWAS) could serve as an effective complementary approach to genome-wide association studies, enhancing our ability to identify genes linked to traits like diseases and elucidating the intricate regulatory interactions among them (161). For lncRNAs, the genetic association signal about transcript abundance within a particular tissue can be juxtaposed against the signal associated with a specific disease, thereby facilitating the validation of the disease’s causal relationship. Employing these strategies enables the identification of lncRNAs that hold the greatest relevance for experimental investigation. Ideally, these lncRNAs should be tested using humanized models to gain a deeper insight into their underlying mechanisms of action (160, 162).

Existing tumor markers have poor sensitivity and specificity. However, reliable biomarkers for predicting the prognosis of patients with cancer are still lacking. Considering the high stability of lncRNAs in blood and their resistance to nuclease degradation, circulating lncRNAs appear to be reliable and promising prognostic biomarkers. Circulating lncRNAs have been used as reliable diagnostic and prognostic markers in various cancers, including liver, colorectal, gastric, and prostate cancers (163). In particular, the available biomarkers for predicting the response to immunotherapy are limited. Given the role of lncRNAs in mediating the immunosuppressive TME, circulating lncRNAs are expected to be complementary markers for predicting the efficacy of immunotherapy. Further studies are needed to explore the feasibility of using lncRNAs as prognostic markers in clinical practice.

## 7 Conclusions

Tumors initiate metabolic reprogramming to support their rapid proliferation, altering the TME. This triggers competition for nutrients between tumor and immune cells, leading to metabolic changes in immune cells that promote an immunosuppressive microenvironment and tumor immune evasion. LncRNAs impact TME remodeling via these metabolic shifts. Targeted lncRNA therapy aims to overcome immunotherapy resistance and drug tolerance in tumor patients, presenting a novel anti-tumor strategy.
