# Correction: LncRNAs in tumor metabolic reprogramming and tumor microenvironment remodeling

**DOI:** 10.3389/fimmu.2026.1800183

**Published:** 2026-02-13

**Authors:** Jianhang Jiao, Yangzhi Zhao, Qimei Li, Shunzi Jin, Zhongshan Liu

**Affiliations:** 1 Department of Orthopedics, The Second Affiliated Hospital of Jilin University, Changchun, Jilin, China; 2 Department of Hematology, The First Hospital of Jilin University, Changchun, China; 3 Department of Radiation Oncology, The Second Affiliated Hospital of Jilin University, Changchun, China; 4 NHC Key Laboratory of Radiobiology, Jilin University, Changchun, China

**Keywords:** lncRNAs, metabolic reprogramming, tumor microenvironment remodeling, tumor immunity, tumor immunotherapy

In the published article, there was a mistake in the **Funding** statement. The incorrect statement read: “the author(s) declare that financial support was received for the research, authorship, and/or publication of this article. This work was supported by Department of Science and Technology of Jilin Province (Grant No. YDZJ202301ZYTS086); Department of Health Science and Technology of Jilin Province (Grant No. 2022LC115); Department of Science and Technology of Jilin Province (Grant No. YDZJ202201ZYTS135); Bethune Plan of Jilin University (Grant No. 2023B08)”. The correct **Funding** statement is: “The author(s) declare that financial support was received for the research, authorship, and/or publication of this article. This work was supported by Department of Science and Technology of Jilin Province (Grant No. YDZJ202301ZYTS086); Department of Health Science and Technology of Jilin Province (Grant No. 2022LC115); Bethune Plan of Jilin University (Grant No. 2023B08)”.

The original version of this article has been updated.

